# Association of dietary total antioxidant capacity with all-cause and cardiovascular mortality in patients with chronic kidney disease: based on two retrospective cohort studies of NHANES

**DOI:** 10.1080/0886022X.2023.2205950

**Published:** 2023-05-31

**Authors:** Yue Li, Gui-Chen Ling, Rui-Bin Ni, Shi-Hao Ni, Shu-Ning Sun, Xin Liu, Jian-Ping Deng, Xiao-Lu Ou-Yang, Jin Li, Shao-Xiang Xian, Ling-Jun Wang, Tao-Chun Ye, Lu Lu

**Affiliations:** a The First Affiliated Hospital, Guangzhou University of Chinese Medicine, Guangzhou, China; b Lingnan Medical Research Center, Guangzhou University of Chinese Medicine, Guangzhou, China; c University Key Laboratory of Traditional Chinese Medicine Prevention and Treatment of Chronic Heart Failure, Guangdong Province, Guangzhou, China; d The Fourth Clinical Medical College of Guangzhou University of Chinese Medicine, Shenzhen, China; e The Second Clinical Medical College of Guangzhou University of Chinese Medicine, Guangzhou, China

**Keywords:** Dietary total antioxidant capacity, mortality, chronic kidney disease, NHANES, vitamin C equivalent antioxidant capacity, component dietary antioxidant index

## Abstract

**Background:**

The relationship between dietary total antioxidant capacity (DTAC) and death risk among CKD populations remains unclear.

**Methods:**

Based on vitamin C equivalent antioxidant capacity (VCEAC) and the component dietary antioxidant index (CDAI) indices, we analyzed two cohorts to investigate the association of DTAC with all-cause and CVD mortality in CKD patients using data from National Health and Nutrition Examination Survey (2007–2018). VCEAC (*n* = 6330) and CDAI (*n* = 6300) cohorts with mortality follow-up data available through 2018 were included. Cox models with restricted cubic splines was used to model the nonlinear association between VCEAC/CDAI and outcomes in CKD patients.

**Results:**

Our results showed L-shaped associations of DTAC with all-cause mortality among individuals with CKD stages 1–2 in both cohorts. Compared to the lowest quartile, higher dietary total antioxidant intake was associated with lower all-cause mortality risks among CKD stages 1–2 after adjustment for covariates, with HRs (95%CI) of 1.00, 0.91 (0.71,1.17), 0.69 (0.53,0.90), and 0.70 (0.54,0.91) in VCEAC, and similar respective estimate trends in CDAI. After sensitivity and subgroup analyses, there were no benefits for patients with stage 3–5 CKD or albuminuria. Mediation analysis revealed that the proportions mediated in both cohorts were less consistent.

**Conclusions:**

Moderate dietary total antioxidants intake has potential benefits for early-stage CKD patients. However, further evidence is needed to confirm whether patients with worsening CKD can benefit in the long term.

## Introduction

Chronic kidney disease (CKD) is a major public health problem worldwide, with an estimated projection to be the fifth leading cause of death globally by 2040 [1]. Immediate efforts are therefore warranted to control the disease with interventions that target modifiable risk factors, including diet. Patients with CKD may be at risk for multiple nutritional and metabolic abnormalities involving protein-energy malnutrition and micronutrient deficiencies, protein-energy wasting, and electrolyte disturbances in all stages [2].

Oxidative stress plays a pivotal role in the pathogenesis of renal disorders, including acute kidney injury and CKD [3]. An overabundance of reactive oxygen species disrupts redox homeostasis, leading to inflammation, apoptosis, and mitochondrial dysfunction with potentially devastating consequences for renal function [4]. Kidney cells possess an intrinsic defense mechanism to mitigate the deleterious effects of oxidative stress. A key component of this system is Nuclear factor erythroid 2-related factor 2 (NRF2), which serves a critical role in safeguarding cells from oxidative damage [5]. This may reduce the harmful effects of oxidative stress on the kidneys, potentially slowing or even reversing damage [6,7]. Notably, bioactive compounds such as (-)-Epicatechin, epigallocatechin-3-gallate, and selenium can boost NRF2-related antioxidant pathway [8–10]. Certain dietary patterns have also been found to enhance these defenses, making them an important consideration for individuals seeking to improve their kidney health [11].

Considering the whole diet, dietary total antioxidant capacity (DTAC) considers the cumulative effect of antioxidants as a commonly used parameter for assessing antioxidant capacity from the diet [12]. The component dietary antioxidant index (CDAI) and vitamin C equivalent antioxidant capacity (VCEAC) are two indices to calculate the quantitative value of DTAC for dietary intake [13–15]. Evidence from cohort studies reported that healthy dietary patterns or adequate intake of micronutrients might lower the risk of CKD [16–18]. DTAC may be involved in predicting the prognosis of CKD, but this field needs more robust evidence.

This study explored the complex relations between DTAC and all-cause or CVD mortality using data from the National Health and Nutrition Examination Survey (NHANES) (2007–2018).

## Materials and methods

### Study population

**Figure 1.**
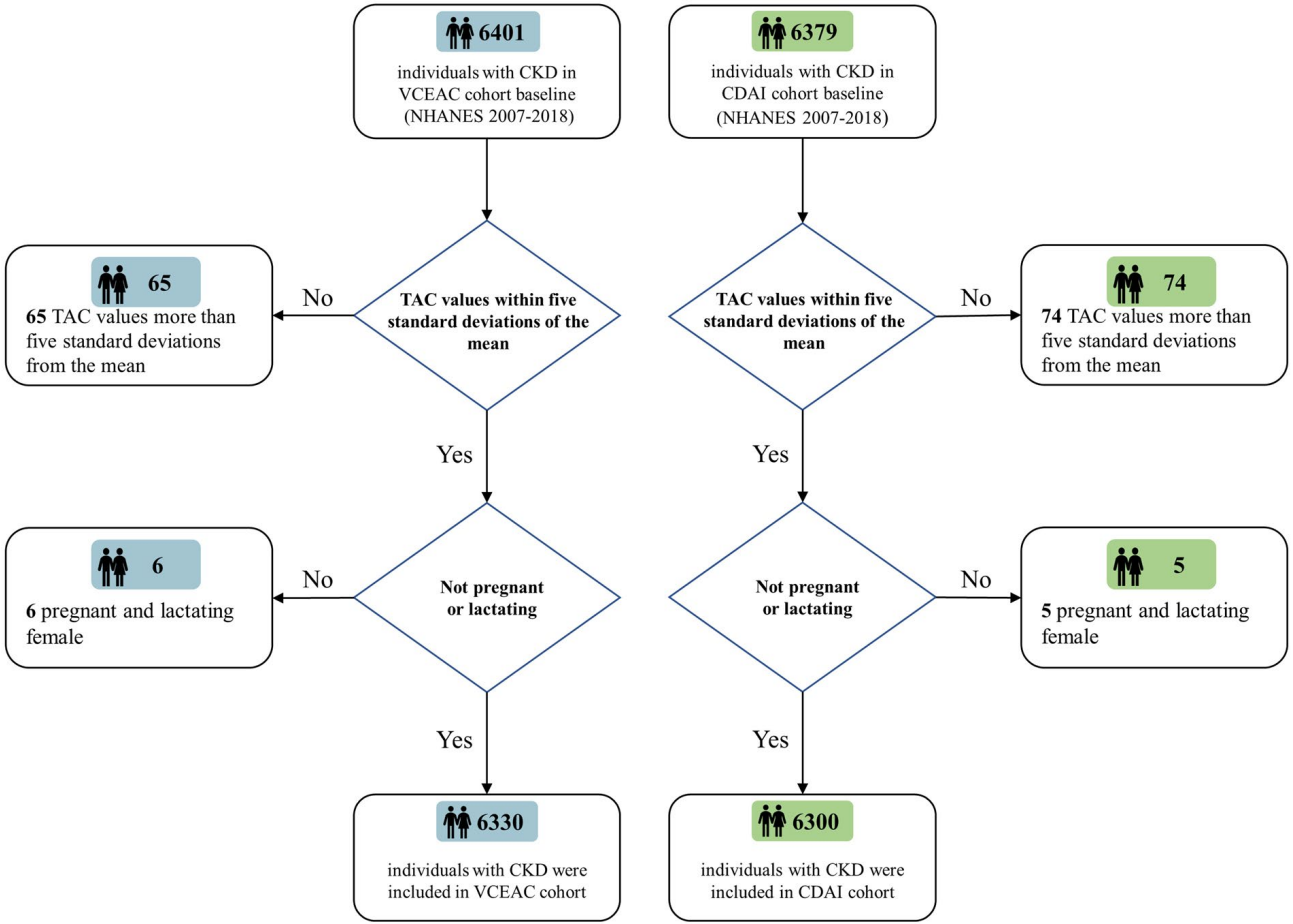
Flowchart for the selection of the study population, NHANES (2007–2018). A total of 6401 participants for VCEAC cohort and 6379 for CDAI cohort were separately eligible for criteria. After the exclusion of those pregnant or lactating women, and those with TAC values more than five standard deviations from the mean, 6330 participants with CKD were included in the final VECAC cohort analysis and 6300 participants with CKD were included in the final CDAI cohort analysis.

The NHANES used a complex, multistage, probability sampling design to monitor trends in the health and nutritional status of the US population. It is conducted by the Centers for Disease Control and Prevention (CDC). The following criteria were considered in selecting patients in cohorts [1]: aged >18 years who completed at least 1 dietary antioxidant recall [2]; estimated glomerular filtration rate (eGFR) <90 mL/min/1.73 m^2^ using the CKD-EPI equations or urinary albumin >200 mg/L; and complete mortality follow-up status record. We separately included 6401 participants for the VCEAC cohort and 6379 for the CDAI cohort at the baseline survey from continuous NHANES (2007–2018) datasets. Simultaneously, we excluded participants who met any of the exclusion criteria [1]: pregnant or lactating women and [2] TAC values more than five standard deviations from the mean. Finally, a total of 6330 adult candidates remained in our cohort for VCEAC analysis and a total of 6300 participants remained in the CDAI analysis cohort (Figure 1).

### Dietary information

Dietary information from NHANES was obtained through two 24-h recall interviews and a food-frequency questionnaire (FFQ) [19]. The United States Department of Agriculture (USDA) food composition databases and Nutrient Database for Dietary Studies (FNDDS) have been provided a unique research tool to evaluate dietary antioxidants and micronutrient intake values [20]. In the present study, antioxidant nutrients include 29 flavonoids, 5 types of carotenoids, vitamin A, vitamin C, vitamin E, and minerals (magnesium, selenium, and zinc) as determined by the daily consumption of each selected food item and supplement. In addition, the protein intake, energy intake, carbohydrate intake, dietary fiber intake, and total fat intake were included in this study.

### Calculation of the CDAI and evaluation of dietary total antioxidant capacity

The dietary total antioxidant capacity was determined by the vitamin C equivalent antioxidant capacity (VCEAC) and the component dietary antioxidant index (CDAI).

For VCEAC calculation, the antioxidant capacity of vitamin C, 29 types of flavonoids, 5 types of carotenoids, vitamin A, and vitamin E was analyzed by the 2,2′-azino-bis(3-ethylbenzthiazoline) 6-sulphonic acid (ABTS) method as previously described [13–15]. Then, a standard curve linearly fitted the association between the antioxidant capacity of vitamin C and the optical density value. The VCEAC of other antioxidant dietary intakes was determined by the standard vitamin C curve and then summed. The value of VCEAC represents that the antioxidant capacity of the 100 mg assigned diet equals the quality of vitamin C. Because the antioxidant capacity of minerals (magnesium, selenium, and zinc) cannot be tested by the ABTS method, we excluded them when calculating VCEAC.

For CDAI calculation, the intake of selected antioxidants (including 29 types of flavonoids, 5 types of carotenoids, vitamin A, vitamin C, vitamin E, and 3 types of minerals as above) was normalized by the zero-mean method [(daily intake of antioxidant - mean intake of antioxidant)/standard deviation]. Then the total information of all antioxidant categories was summed as previously described [13,21].

The values of VCEAC and CDAI were adjusted for energy *via* the residual method. More detailed methods can be found in the Supplementary Methods.

### Assessment of other covariates

Potential confounding factors of the relationship between DTAC and CKD progression were considered covariates. Included covariates were as follows: age, ethnicity, body mass index (BMI), sex, education, income, smoking, alcohol, energy intake, protein, total dietary fat, carbohydrate, dietary fiber, total fat, and disease histories of diabetes mellitus, hypertension, cancer, cardiovascular disease. The estimated glomerular filtration rate (eGFR) was calculated by the Chronic Kidney Disease Epidemiology Collaboration (CKD-EPI) equation and urine albumin creatinine ratio [22]. In addition, physical activity was converted into the total metabolic equivalent of task (MET) based on the Global Physical Activity Questionnaire (GPAQ) [23,24].

### Outcome

Outcome measures were all‐cause and cardiovascular mortality through linkage to the National Death Index database matching death certificate records. The public-use Linked Mortality File is provided by the National Center for Health Statistics (NCHS), and mortality follow-up data are available through 2018 [25]. CVD mortality was defined by the recorded underlying cause of death on the death certificates (ICD-10 codes I00–I09, I11, I13, and I20–I51) or cerebrovascular disease (ICD-10 codes I60–I69). Overall, 1339 deaths (including 422 CVD-related records) were recorded in our cohort.

### Descriptive statistics

The baseline characteristics of the participants were described by quartiles of the VCEAC and CDAI. Continuous variables are reported as the means and standard deviations (SD) and were compared using one-way ANOVA. Categorical variables are denoted as numbers (percentages), and statistical comparisons were made with the chi-squared test. A two-sided *p* value <.05 was considered statistically significant.

### Cox and restricted cubic spline regression

Cox regression models were used to evaluate the association of VCEAC/CDAI with all-cause and CVD mortality. The hazard ratios and 95% CIs were determined in each quartile using the lowest quartile as a reference. Model 1 was adjusted for demographic variables (age, sex, ethnicity, income, and education level). Model 2 was further adjusted for dietary information and lifestyle (potassium, protein intake, carbohydrate intake, dietary fiber intake, total fat intake, alcohol intake, smoking, and MET-PA, and total energy intake was adjusted *via* the residual method). Model 3 was further adjusted for health conditions (diabetes, hypertension, CVD and cancer, urine albumin, eGFR, BMI).

Restricted cubic spline regression was used to model the nonlinear association between VCEAC/CDAI and outcomes in CKD patients. The first quartile of VCEAC/CDAI was set as a reference as it yields a hazard ratio equal to zero. The first and last knots were placed at the 0.01 and 0.99 quantiles of dietary total antioxidant intake. The nonlinearity of the association was performed using the methods described by Frank Harrell [26].

### Subgroup and sensitivity analysis

To confirm the stability of our findings, we also performed a subgroup analysis stratified by sex, age, BMI, diabetes, cancer, and CVD, urinary albumin, and CKD stage. The interaction between subgroups was conducted using B Schneider’s regression models [27]. We also conducted sensitivity analyses and excluded the following participants: those whose cause of death was trauma, those who died after less than two years of follow-up, those who died of malignant neoplasms, and those with mild CKD (1–2 stage).

### Mediation effect analysis

To quantify the mediating effect of each antioxidant, we performed an analysis to investigate the mediating effect of each antioxidant included in the index on the association between DTAC and outcome. We first examined the linear relationship between the intake of each antioxidant intake and the VCEAC/CDAI. Then, the time-dependent outcome and all variables were fitted using the survival regression model. Further details of the average causal mediation effect (ACME) and total effect are provided in Kosuke Imai’s methods [28]. The 95% CI was calculated using the bootstrap method with 2000 replications.

### Statistical tools

Cox regression analyses were performed using the survival R package and R version 3.6.1. A Cox proportional hazards regression model with restricted cubic splines (RCS) was conducted using the RCS R package. We used the R package ‘Publish’ for the subgroup analysis and the R package ‘medication’ for the medicated effect analysis.

## Results

### Participants’ baseline characteristics

A total of 6330 participants were included in the VCEAC cohort (mean age 62.69 years, 52.3% male). The cohort was stratified into quartiles, with approximately 25% of participants in each quartile. Similarly, the CDAI cohort comprised 6300 participants with a mean age of 62.73 years and a male proportion of 52.23%, and was also stratified into quartiles (Supplementary Table 1 and Table 2). In both VCEAC and CDAI cohorts, participants in the highest quartile of DTAC had a higher intake of alcohol, total energy, carbohydrates, dietary fiber, and total fat (*p* < .001). They were also more likely to have a history of cancer (*p* < .001). These individuals had higher income (*p* < .001) and were more likely to be white or of other ethnicity (*p* < .001). Compared to those in the lowest quartile, they had lower rates of smoking and BMI (*p* < .001) in the VCEAC cohort and also had lower rates of smoking, hypertension, diabetes, and cardiovascular disease (*p* < .001) as well as PA_total_MET (*p* = .003) in the CDAI cohort.

### Association between dietary total antioxidant capacity and all-cause/CVD mortality

**Figure 2.**
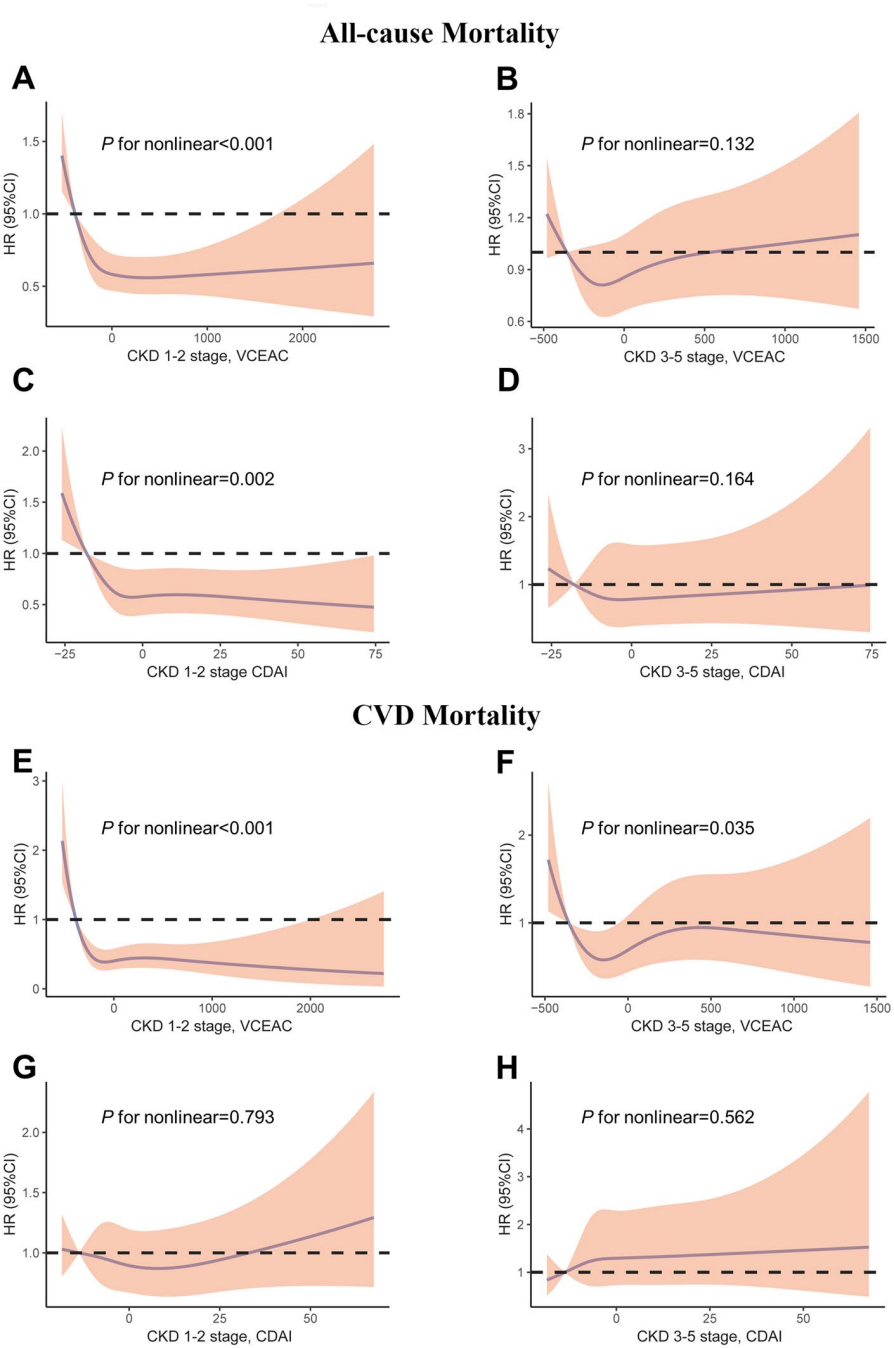
Restricted cubic spline models for the relationship between dietary total antioxidant capacity with all-cause (top) and CVD (bottom) mortality. (A) CKD 1–2 stage, VCEAC and all-cause mortality. (B) CKD 3–5 stage, VCEAC and all-cause mortality (C) CKD 1–2 stage, CDAI and all-cause mortality. (D) CKD 3–5 stage, CDAI and all-cause mortality (E) CKD 1–2 stage, VCEAC and CVD mortality. (F) CKD 3–5 stage, VCEAC and CVD mortality. (G) CKD 1–2 stage, CDAI and CVD mortality. (H) CKD 3–5 stage, CDAI and CVD mortality. Adjusted for age, sex, ethnicity, income, education level, potassium intake, protein intake, carbohydrate intake, dietary fiber intake, total fat intake, alcohol intake, total energy intake *via* the residual method, smoking, MET-PA, diabetes, hypertension, CVD and cancer, urine albumin, eGFR, and BMI. The first quartile of VCEAC/CDAI was used as reference as it yields a hazard ratio of 0. The first and last knots were placed at the 0.01 quantile and 0.99 quantile of DTAC. Shading indicates the 95% confidence interval.

Table 1 shows that higher levels of dietary total antioxidant intake in patients with CKD stages 1–2 were associated with a trend toward a lower risk of all-cause mortality, but the association was likely to be nonlinear. The hazard ratios (95%CI) from the first quartile to the highest quartile were 1.00, 0.91(0.71 to 1.17, *p* = .463), 0.69 (0.53 to 0.90, *p* = .005), and 0.70 (0.54 to 0.91, *p* = .009) in the VCEAC cohort. A consistent result was discovered in the CDAI cohort. Hazard ratios (95%CI) from the first to the fourth quartile of CDAI were 1.00, 0.69 (0.52 to 0.91, *p* = .008), 0.79 (0.60 to 1.05, *p* = .110), and 0.73 (0.53 to 1.00, *p* = .053). However, no associations were observed for all-cause mortality among participants with CKD stages 3–5 and with higher dietary total antioxidant intake. In addition, the association of DTAC with CVD mortality was not significant in the two cohorts (Table 2). Restricted cubic splines were used to visualize and examine the possibility of a nonlinear relation of DTAC with all-cause mortality and CVD mortality in the VCEAC and CDAI cohorts (Figure 2). The results indicate that there was an L-shaped relationship between DTAC and all-cause mortality among patients with CKD stages 1–2, but not among those with CKD stages 3–5, in two cohorts. Similarly, there were nonlinear relationships observed for DTAC and CVD mortality among patients with CKD in the VCEAC cohort, but no evidence of nonlinear associations between DTAC and CVD mortality in the CDAI cohort.

**Table 1.** Association of VCEAC and CDAI with all-cause mortality among patients with CKD in the NHANES (2007–2018).

	VCEAC							CDAI						
	Q1	Q2		Q3		Q4		Q1	Q2		Q3		Q4	
		HR (95% CI)	*p* Value	HR (95% CI)	*p* Value	HR (95% CI)	*p* Value		HR (95% CI)	*p* Value	HR (95% CI)	*p* Value	HR (95% CI)	*p* Value
CKD 1–2 stage														
Model1	Ref	0.86(0.67–1.09)	.203	0.87(0.68–1.12)	.291	0.91(0.70–1.17)	.448	Ref	0.72(0.56–0.91)	.007	0.69(0.53–0.90)	.006	0.66(0.50–0.86)	.002
Model2	Ref	0.88(0.68–1.13)	.303	0.72(0.55–0.93)	.011	0.74(0.57–0.95)	.019	Ref	0.74(0.56–0.98)	.035	0.82(0.62–1.09)	.175	0.78(0.57–1.07)	.122
Model3	Ref	0.91(0.71–1.17)	.463	0.69(0.53–0.90)	.005	0.70(0.54–0.91)	.009	Ref	0.69(0.52–0.91)	.008	0.79(0.60–1.05)	.110	0.73(0.53–1.00)	.053
CKD 3–5 stage														
Model1	Ref	0.78(0.58–1.04)	.091	0.75(0.54–1.03)	.072	0.74(0.53–1.02)	.068	Ref	0.73(0.55–0.96)	.024	0.71(0.53–0.94)	.018	0.73(0.55–0.97)	.028
Model2	Ref	0.76(0.55–1.05)	.099	0.63(0.47–0.87)	.004	0.83(0.61–1.14)	.246	Ref	0.80(0.61–1.07)	.130	0.75(0.55–1.02)	.066	0.87(0.63–1.20)	.392
Model3	Ref	0.75(0.54–1.04)	.081	0.61(0.44–0.83)	.002	0.81(0.59–1.12)	.210	Ref	0.89(0.66–1.19)	.432	0.81(0.60–1.10)	.186	0.93(0.68–1.29)	.678

VCEAC: Vitamin C Equivalent Antioxidant Capacity; CDAI: Component Dietary Antioxidant Index; HR: hazard ratio; CI: confidence interval; Q: quartile.

*Note:* Model1: adjusted for age, sex, ethnicity, income, and education level. Model 2: Model1 + potassium, protein, carbohydrates, dietary fiber, total fat, alcohol intake, smoking, and MET-PA, and total energy intake was adjusted *via* the residual method; Model3: Model2 + diabetes, hypertension, CVD and cancer, urine albumin, eGFR, BMI.

**Table 2.** Association of VECAC and CDAI with CVD mortality among patients with CKD in the NHANES (2007–2018).

	VCEAC							CDAI						
	Q1	Q2		Q3		Q4		Q1	Q2		Q3		Q4	
		HR (95% CI)	*p* Value	HR (95% CI)	*p* Value	HR (95% CI)	*p* Value		HR (95% CI)	*p* Value	HR (95% CI)	*p* Value	HR (95% CI)	*p* Value
CKD 1–2 stage														
Model1	Ref	0.74(0.49–1.10)	.135	0.77(0.52–1.13)	.184	0.83(0.55–1.25)	.364	Ref	1.01(0.68–1.51)	.957	1.12(0.72–1.74)	.620	1.11(0.70–1.76)	.661
Model2	Ref	0.76(0.51–1.13)	.172	0.55(0.35–0.87)	.010	0.69(0.45–1.04)	.077	Ref	0.85(0.51–1.40)	.520	0.75(0.44–1.27)	.278	0.84(0.48–1.49)	.559
Model3	Ref	0.82(0.54–1.25)	.352	0.51(0.32–0.81)	.004	0.64(0.40–1.01)	.057	Ref	0.82(0.50–1.36)	.444	0.73(0.43–1.24)	.244	0.82(0.46–1.47)	.501
CKD 3–5 stage														
Model1	Ref	0.95(0.53–1.71)	.858	1.05(0.56–1.97)	.884	1.10(0.56–2.15)	.791	Ref	1.36(0.80–2.32)	.251	1.64(0.97–2.79)	.066	1.64(0.97–2.77)	.066
Model2	Ref	1.04(0.55–1.96)	.902	0.87(0.51–1.48)	.604	1.01(0.55–1.87)	.969	Ref	1.14(0.68–1.90)	.615	1.44(0.84–2.48)	.186	1.29(0.73–2.27)	.381
Model3	Ref	1.08(0.55–2.13)	.813	0.79(0.46–1.39)	.418	1.01(0.50–2.02)	.981	Ref	1.27(0.75–2.13)	.370	1.58(0.92–2.71)	.097	1.36(0.79–2.36)	.267

VCEAC: Vitamin C Equivalent Antioxidant Capacity; CDAI: Component Dietary Antioxidant Index; HR: hazard ratio; CI: confidence interval; Q: quartile.

*Notes:* Model1: adjusted for age, sex, ethnicity, income, and education level. Model 2: Model1 + potassium, protein, carbohydrates, dietary fiber, total fat, alcohol intake, smoking, and MET-PA, and total energy intake was adjusted *via* the residual method; Model3: Model2 + diabetes, hypertension, CVD and cancer, urine albumin, eGFR, BMI.

### Subgroup and sensitivity analyses

**Figure 3.**
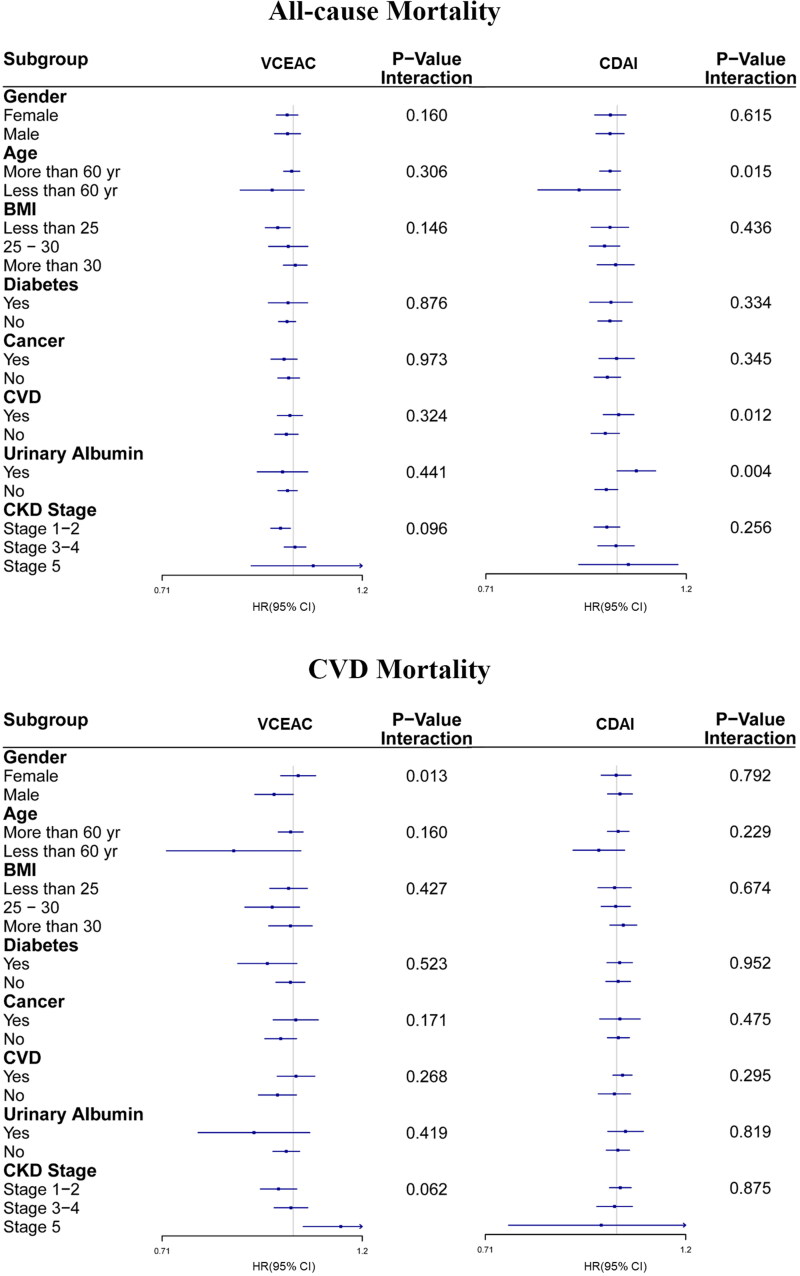
Subgroup analyses of multi-variable adjusted association of VCEAC or CDAI with all-cause (top) and CVD (bottom) mortality in the NHANES (2007–2018), stratified by selected patients’ characteristics. (A) VCEAC and CDAI with all-cause mortality. (B) VCEAC and CDAI with CVD mortality. Abbreviations: HRs: hazard ratios; CI: confidence intervals; CVD: cardiovascular disease; CDAI: Component Dietary Antioxidant Index*;* VCEAC: Vitamin C Equivalent Antioxidant Capacity. Adjusted for ethnicity, income, and education level, smoking status, potassium intake, protein intake, alcohol intake, total dietary fat intake, carbohydrate intake, and total energy intake was adjusted *via* the residual method, BMI, MET-PA, with exception of stratifying factors.

We performed stratified analyses by the patients’ characteristics (Figure 3) and found that every 5-unit increase in CDAI or 100-unit increase in VCEAC was associated with an increased risk of all-cause mortality or CVD mortality. In the VCEAC cohort, stratified analysis was not found to be statistically significant for all-cause mortality risk. However, male individuals with higher DTAC levels had a lower risk of CVD mortality than female (0.95 [95% CI, 0.90–1.00] versus (1.01 [95% CI, 0.97–1.06]; *p* for interaction = .013). There was a trend toward lower CVD mortality risk (*p* for interaction=.062) in the CKD stages 1–2 group, but the all-cause and CVD mortality risks increased incrementally with worsening CKD (*p* for interaction = .096). In the CDAI cohort subgroup analysis, individuals with a history of cardiovascular disease had higher hazard ratios of all-cause mortality than those without (1.00 [95% CI, 0.96–1.05] versus (0.97 [95% CI, 0.93–1.01]; *p* for interaction = .012).We also found that individuals with urinary albumin had marginally higher all-cause mortality than those with no urinary albumin (*p* = .004), but no significant differences in CVD mortality risk were found. Our sensitivity analyses (Supplementary Table 3) confirmed our results. After excluding participants with mild CKD (1–2 stage), DTAC was still associated with an increased HR for all-cause mortality and CVD mortality.

### Mediation analysis with independent antioxidant vitamins/minerals

We conducted a mediation analysis to examine the indirect effects of independent antioxidant vitamins/minerals on the associations between DTAC and mortality outcomes. The results are presented in Tables 3 and 4. In the VCEAC cohort study, we estimated the proportion of the average causal mediated effect (ACME) of independent antioxidant vitamins/minerals on all-cause mortality. We found that vitamin E, flavones, beta-cryptoxanthin and anthocyanidins accounted for 32.28%, 25.67%, 21.90% and 8.23% of the total effect, respectively. The mediation proportion was similar for CVD mortality. In the CDAI cohort study, we calculated the total effect and ACME of magnesium and vitamin E on all-cause mortality. The total effect was 3.43(1.02–5.88) for magnesium and 3.23(1.05–5.70) for vitamin E, while the ACME was 1.93(1.0–3.04) for magnesium and 0.80(0.07–1.59) for vitamin E. Magnesium and vitamin E mediated the association by 55.94% (*p* = .008) and 24.21% (*p* = .040), respectively. We did not detect any mediation for CVD mortality.

**Table 3.** Mediation analysis of VCEAC with mortality of CKD.

	All-cause mortality				CVD mortality			
	Total effects (95%CI)	ACME (95%CI)	Mediation proportion (%)	*p* Value	Total effects (95%CI)	ACME (95%CI)	Mediation proportion (%)	*p*Value
Vitamin A	0.12(0.03–0.22)	−0.02(−0.07–0.03)	–	.43	0.12(0.03–0.22)	−0.02(−0.08–0.03)	–	.408
Vitamin C	0.12(0.03–0.21)	0.01(−0.02–0.03)	–	.67	0.12(0.02–0.21)	0.01(−0.02–0.03)	–	.676
Vitamin E	0.12(0.02–0.22)	0.04(0.01–0.07)	32.28	.01	0.12(0.03–0.21)	0.04(0.01–0.07)	32.47	.012
Beta-carotene	0.12(0.02–0.21)	−0.02(−0.19–0.14)	–	.84	0.12(0.02–0.21)	−0.02(−0.18–0.15)	–	.802
Alpha-carotene	0.11(0.02–0.20)	−0.08(−0.19–0.04)	–	.20	0.11(0.02–0.21)	−0.07(−0.18–0.04)	–	.208
Beta-cryptoxanthin	0.12(0.03–0.22)	0.03(0.00–0.06)	21.90	.03	0.13(0.03–0.23)	0.03(0.00–0.06)	22.01	.020
Lycopene	0.12(0.03–0.21)	0.01(−0.02–0.04)	–	.61	0.11(0.03–0.21)	0.01(−0.02–0.04)	–	.536
Lutein_zeaxanthin	0.12(0.03–0.22)	−0.01(−0.06–0.04)	–	.71	0.12(0.03–0.22)	−0.01(−0.06–0.04)	–	.732
Carotenoids	0.12(0.03–0.22)	0.01(−0.02–0.03)	–	.59	0.12(0.03–0.21)	0.01(−0.02–0.03)	–	.556
Isoflavones	0.12(0.03–0.21)	0.00(0.00–0.00)	–	.74	0.12(0.03–0.21)	0.00(0.00–0.00)	–	.770
Anthocyanidins	0.12(0.03–0.22)	0.01(0.00–0.02)	8.23	.03	0.13(0.03–0.22)	0.01(0.00–0.02)	8.21	.026
Flavan-3-ols	0.12(0.03–0.21)	0.00(−0.02–0.03)	–	.79	0.12(0.02–0.21)	0.00(−0.02–0.02)	–	.844
Flavanones	0.12(0.03–0.21)	0.00(−0.01–0.00)	–	.66	0.12(0.03–0.21)	0.00(−0.01–0.00)	–	.660
Flavones	0.12(0.03–0.22)	0.03(0.00–0.07)	25.67	.04	0.12(0.03–0.22)	0.03(0.01–0.07)	27.12	.046
Flavonols	0.12(0.03–0.21)	0.02(−0.01–0.05)	–	.23	0.12(0.03–0.22)	0.02(−0.01–0.05)	–	.228

ACME: average causal mediation effects.

**Table 4.** Mediation analysis of CDAI with mortality of CKD.

	All-cause mortality				CVD mortality			
	Total effects (95%CI)	ACME (95%CI)	Mediation proportion (%)	*p*Value	Total effects (95%CI)	ACME (95%CI)	Mediation proportion (%)	*p* Value
Vitamin C	3.11(0.69–5.34)	0.12(−0.59–0.80)	–	0.710	−0.42(−14.57–12.72)	0.06(−4.04–4.08)	–	0.990
Vitamin E	3.23(1.05–5.70)	0.80(0.07–1.59)	24.21	0.040	0.51(−12.13–14.51)	3.91(−0.13–9.49)	–	0.952
Selenium	3.18(0.90–5.56)	0.00(−0.04–0.04)	–	0.936	−0.01(−13.07–11.80)	−0.09(−0.52–0.24)	–	0.976
Magnesium	3.43(1.02–5.88)	1.93(1.02–3.04)	55.94	0.008	0.30(−13.20–13.53)	5.87(0.37–13.49)	–	0.944
Zinc	3.28(0.93–5.71)	−0.14(−0.36–0.05)	–	0.140	−0.23(−12.91–11.60)	−0.41(−1.70–0.67)	–	0.986
Carotenoids	3.37(1.03–5.96)	−1.76(−4.82–1.16)	–	0.252	−0.48(−13.57–12.11)	−3.15(−20.55–12.65)	–	0.966
Isoflavones	3.06(0.70–5.42)	−0.50(−1.09–−0.01)	–	0.044	0.75(−11.62–13.13)	−2.27(−4.88–−0.53)	–	0.882
Anthocyanidins	3.41(0.87–5.89)	0.96(−0.20–2.15)	–	0.092	−0.28(−12.76–12.57)	2.72(−2.40–9.18)	–	0.960
Flavan-3-ols	3.32(0.96–5.91)	−2.15(−5.12–0.72)	–	0.160	−0.58(−14.16–12.06)	−1.33(−19.10–14.42)	–	0.986
Flavanones	3.16(0.92–5.47)	−0.21(−0.62–0.17)	–	0.308	−0.09(−13.66–13.36)	0.95(−1.48–3.73)	–	0.984
Flavones	3.27(0.95–5.88)	1.05(−0.04–2.29)	–	0.080	0.90(−11.95–14.54)	6.87(−0.42–15.81)	–	0.842
Flavonols	3.13(0.78–5.42)	−0.11(−3.14–2.79)	–	0.942	−0.33(−13.87–12.58)	−4.64(−21.48–11.32)	–	1.000

ACME: average causal mediation effects.

## Discussion

In our study of nationally representative US adults with CKD, we found that moderate consumption of dietary total antioxidant intakes, indicated by the Vitamin C Equivalent Antioxidant Capacity (VCEAC) and the Component Dietary Antioxidant Index (CDAI), was associated with a lower risk for all-cause mortality in individuals with CKD 1–2 stages. However, higher dietary total antioxidant intake may increase the risk of all-cause mortality in patients with CKD stages 3–5 or higher albuminuria. On the other hand, there are some intrinsic differences between the two methods. The VCEAC-based method appeared more predictive of cardiovascular (CVD) mortality risk. Our study found that in the CDAI cohort, up to 55.94% of the association between dietary total antioxidant capacity and mortality was mediated by Magnesium intake. In contrast, the VCEAC cohort was more equally balanced in interpreting the role of individual antioxidant micronutrients.

Adhering to a healthy dietary pattern includes more whole grains, fruits, vegetables, and healthy fats; more fiber, vitamins C and E, and carotenoids; less saturated fat, salt, and processed food; and a lower acid load may help prevent CKD and proteinuria [29]. In our study, dietary factors that may help prevent CKD include total antioxidants in the diet, including vitamins, 29 types of flavonoids, and trace minerals. Micronutrients play a significant role in antioxidant status, and CKD patients have been found to have significantly lower intake of antioxidant-rich foods [30]. Several studies have suggested that individuals with low levels of selenium have impaired renal function, and deficiency of this trace element may contribute to increased oxidative stress and inflammation [31–33]. Previous clinical trials mainly focused on the hemodialysis therapy cohort using antioxidants to demonstrate that the beneficial effects in patients with CKD have been disappointing [34,35], individuals with CKD exhibit diminished antioxidant defenses due to dietary restrictions on high-potassium fruits and vegetables, decreased levels of vitamins C and E, loss of selenium during dialysis, and reduced glutathione (GSH) scavenging capacity. Normally, endogenous or dietary antioxidants safeguard cells against oxidative stress, inflammation, and damage. However, in CKD these defenses are compromised, potentially due to multiple factors contributing to the endogenous overproduction of ROS in CKD patients, making optimal control of ROS challenging to achieve. Supplementation with antioxidants and implementation of other strategies to enhance defenses could aid in decelerating kidney deterioration in CKD and reducing disease progression [36]. Bardoxolone methyl is an orally potent activator of the Nrf2 pathway against oxidative stress and simultaneously attenuates inflammation by inhibiting NF-κB [37]. Promising results from clinical trial reported that an improvement in eGFR was observed following treatment with bardoxolone methyl in CKD patients with type 2 diabetes [38,39]. Moreover, natural compounds possessing anti-inflammatory and antioxidant attributes have exhibited the capacity to decelerate the advancement of kidney disease and mitigate its associated complications. This is achieved by counterbalancing the detrimental impact of oxidative stress and inflammation, which are hallmarks of chronic kidney disease [40,41]. The delicate balance between inadequate micronutrient intake and overconsumption is a major concern for patients with CKD [2].

Our study highlights an L-shaped correlation between dietary total antioxidant intake and CKD 1–2 stage all-cause mortality risks. Meanwhile, the subgroup and sensitivity analysis observed more benefit for CKD stages 1–2. However, patients with CKD stages 3–5 or albuminuria did not have long-term survival benefits from higher dietary total antioxidant intake and a trend toward an increased risk of all-cause mortality, indicating that dietary intake and recommendations vary depending on the CKD stage. Similarly, a Korean study cohort suggested that inadequate micronutrient intake among the general population may confer an increased risk of CKD stage 3B and over [16]. However, a case–control study reported that a correlation between DTAC and CKD in patients with type 2 diabetes was not obvious [42]. In addition, a RaNCD cohort study showed an inverse and strong association between DTAC and renal function [43]. These results differ from our findings. Another interesting result from the subgroup analysis in our CDAI cohort study indicates that higher dietary total antioxidant intake elevated the risk of all-cause mortality for those with previous cardiovascular events. CKD is an independent risk factor for cardiovascular diseases and is entwined through hormonal mechanisms [44]. Moreover, intestinal dysbiosis, impaired barrier function, and reduced kidney clearance of bacterial byproducts may increase cardiovascular risk in CKD patients [45]. A similar adverse cardiovascular event was observed in a phase III BEACON clinical trial [46], and additional posthoc analyses suggested that bardoxolone methyl promoted acute sodium and volume retention and increased blood pressure through the endothelin pathway precipitating heart failure in an at-risk population [47]. In advanced stages of CKD, the accumulation of waste and insufficient fiber intake can alter the gut microbiome, leading to dysbiosis [48]. Dysbiosis can increase levels of toxic compounds such as TMAO, indoxyl sulfate, and p-cresyl sulfate, contributing to inflammation, oxidative stress, and kidney disease progression [49,50]. Elevated TMAO and other uremic solutes in CKD patients are also linked to increased cardiovascular risk and are being investigated as therapeutic targets [51]. These metabolites, which are normally cleared by the kidneys, can cause tissue damage as kidney function declines. By implementing dietary modifications, it may be feasible to partially restore a healthy balance in the gut microbiome and decrease levels of toxic metabolites in individuals with advanced CKD [52]. Early intervention through dietary total antioxidant intake may potentially delay the progression of CKD.

Our study found that the proportion of mediation by the mediator (individual antioxidant micronutrients) for the association between DTAC and mortality varied considerably in the two cohorts. Moreover, the VCEAC indices are likely more relevant to the projections for CVD mortality. The nature of the methodology may partly explain the heterogeneity in the study results. The VCEAC indices exhibit a more equalized proportion distribution in individual antioxidant micronutrients, although they did not cover the antioxidant capacity of minerals (magnesium, selenium, and zinc). The balanced mediation proportion indicated that DTAC was contributed by multiple sources of antioxidants. The most significant contributor was magnesium intake in the CDAI indices, accounting for 55.94% of the total antioxidant intake. Of note is that observational studies have shown that lower serum magnesium levels are associated with poorer survival in CKD cohorts [53–55]. In contrast, the VCEAC cohort was more equally balanced in interpreting the role of individual antioxidant micronutrients. Thus, DTAC is needed to further rigorous scientific evaluation strategies.

The major strength of our study is the use of the two most common methods (VCEAC and CDAI) to comprehensively evaluate the complex relations of DTAC with all-cause mortality and CVD mortality. The association between DTAC and all-cause mortality was consistent in the VCEAC and CDAI cohorts from well-established NHANES datasets. In addition, we also conducted subgroup and sensitivity analyses and mediation analyses to show the robustness of the findings and evaluated individual antioxidant micronutrients. Nevertheless, our study also has several limitations. First, data regarding dietary total antioxidant intake were assessed by questionnaires. Therefore, recall bias may lead to inaccuracy. Furthermore, due to some missing ICD-10 codes, underestimating cardiovascular death events can increase the chance of type II errors. Finally, this study performed *post hoc* and exploratory subgroup analyses without *a priori* sample size calculations and should therefore be interpreted cautiously.

## Conclusions

Our study suggests that moderate dietary total antioxidant intake may have beneficial effects on patients with earlier stages of CKD. However, further evidence is needed to determine the impact on patients with advanced stages of CKD.

## Ethical approval

The ethical approval are available in NHANES-NCHS Research Ethics Review Board Approval (www.cdc.gov/nchs/nhanes/irba98.htm).

## Supplementary Material

Supplemental Material

## Data Availability

The data that support the findings of this study are openly available in NHANES at http://www.cdc.gov/nhanes. Additional data are available from the corresponding author on reasonable request.

## References

[1] Foreman KJ, Marquez N, Dolgert A, et al. Forecasting life expectancy, years of life lost, and all-cause and cause-specific mortality for 250 causes of death: reference and alternative scenarios for 2016-40 for 195 countries and territories. Lancet. 2018;392(10159):1–11. 30340847 10.1016/S0140-6736(18)31694-5 PMC6227505

[2] MacLaughlin HL, Friedman AN, Ikizler TA. Nutrition in kidney disease: core curriculum 2022. Am J Kidney Dis. 2022;79(3):437–449. 34862042 10.1053/j.ajkd.2021.05.024

[3] Duni A, Liakopoulos V, Roumeliotis S, et al. Oxidative stress in the pathogenesis and evolution of chronic kidney disease: untangling ariadne’s thread. Int J Mol Sci. 2019;20(15):3711. 31362427 10.3390/ijms20153711 PMC6695865

[4] Okamura DM, Pennathur S. The balance of powers: redox regulation of fibrogenic pathways in kidney injury. Redox Biol. 2015;6:495–504. 26448394 10.1016/j.redox.2015.09.039 PMC4600846

[5] Nezu M, Suzuki N, Yamamoto M. Targeting the KEAP1-NRF2 system to prevent kidney disease progression. Am J Nephrol. 2017;45(6):473–483. 28502971 10.1159/000475890

[6] Stenvinkel P, Chertow GM, Devarajan P, et al. Chronic inflammation in chronic kidney disease progression: role of Nrf2. Kidney Int Rep. 2021;6(7):1775–1787. 34307974 10.1016/j.ekir.2021.04.023 PMC8258499

[7] Lu Y, Sun Y, Liu Z, et al. Activation of NRF2 ameliorates oxidative stress and cystogenesis in autosomal dominant polycystic kidney disease. Sci Transl Med. 2020;12(554):eaba3613. 10.1126/scitranslmed.aba3613 32727915

[8] Schwarz M, Lossow K, Kopp JF, et al. Crosstalk of Nrf2 with the trace elements selenium, iron, zinc, and copper. Nutrients. 2019;11(9):2112. 31491970 10.3390/nu11092112 PMC6770424

[9] Huang Z, Jing X, Sheng Y, et al. (-)-epicatechin attenuates hepatic sinusoidal obstruction syndrome by inhibiting liver oxidative and inflammatory injury. Redox Biol. 2019;22:101117. 30822691 10.1016/j.redox.2019.101117 PMC6395886

[10] Mohan T, Narasimhan KKS, Ravi DB, et al. Role of Nrf2 dysfunction in the pathogenesis of diabetic nephropathy: therapeutic prospect of epigallocatechin-3-gallate. Free Radic Biol Med. 2020;160:227–238. 32768570 10.1016/j.freeradbiomed.2020.07.037

[11] Mafra D, Borges NA, Lindholm B, et al. Food as medicine: targeting the uraemic phenotype in chronic kidney disease. Nat Rev Nephrol. 2021;17(3):153–171. 32963366 10.1038/s41581-020-00345-8

[12] Nascimento-Souza MA, Paiva PG, Martino HSD, et al. Dietary total antioxidant capacity as a tool in health outcomes in middle-aged and older adults: a systematic review. Crit Rev Food Sci Nutr. 2018;58(6):905–912. 27646047 10.1080/10408398.2016.1230089

[13] Wright ME, Mayne ST, Stolzenberg-Solomon RZ, et al. Development of a comprehensive dietary antioxidant index and application to lung cancer risk in a cohort of male smokers. Am J Epidemiol. 2004;160(1):68–76. 15229119 10.1093/aje/kwh173

[14] Floegel A, Kim DO, Chung SJ, et al. Development and validation of an algorithm to establish a total antioxidant capacity database of the US diet. Int J Food Sci Nutr. 2010;61(6):600–623. 20377495 10.3109/09637481003670816

[15] Kim DO, Lee CY. Comprehensive study on vitamin C equivalent antioxidant capacity (VCEAC) of various polyphenolics in scavenging a free radical and its structural relationship. Crit Rev Food Sci Nutr. 2004;44(4):253–273. 15462129 10.1080/10408690490464960

[16] Lee J, Oh KH, Park SK. Dietary micronutrients and risk of chronic kidney disease: a cohort study with 12 year Follow-Up. Nutrients. 2021;13(5):1517. 33946331 10.3390/nu13051517 PMC8145051

[17] Farhadnejad H, Asghari G, Mirmiran P, et al. Micronutrient intakes and incidence of chronic kidney disease in adults: Tehran lipid and glucose study. Nutrients. 2016;8(4):217. 27104561 10.3390/nu8040217 PMC4848686

[18] Hu EA, Coresh J, Anderson CAM, et al. Adherence to healthy dietary patterns and risk of CKD progression and all-cause mortality: findings from the CRIC (chronic renal insufficiency cohort) study. Am J Kidney Dis. 2021;77(2):235–244. 32768632 10.1053/j.ajkd.2020.04.019 PMC7855760

[19] Ahluwalia N, Dwyer J, Terry A, et al. Update on NHANES dietary data: focus on collection, release, analytical considerations, and uses to inform public policy. Adv Nutr. 2016;7(1):121–134. 26773020 10.3945/an.115.009258 PMC4717880

[20] Ahuja JK, Moshfegh AJ, Holden JM, et al. USDA food and nutrient databases provide the infrastructure for food and nutrition research, policy, and practice. J Nutr. 2013;143(2):241s–249s. 23269654 10.3945/jn.112.170043

[21] Yu YC, Paragomi P, Wang R, et al. Composite dietary antioxidant index and the risk of colorectal cancer: findings from the Singapore Chinese Health Study. Int J Cancer. 2022;150(10):1599–1608. 35001362 10.1002/ijc.33925 PMC8930521

[22] Levey AS, Stevens LA, Schmid CH, et al. A new equation to estimate glomerular filtration rate. Ann Intern Med. 2009;150(9):604–612. 19414839 10.7326/0003-4819-150-9-200905050-00006 PMC2763564

[23] Cleland CL, Hunter RF, Kee F, et al. Validity of the global physical activity questionnaire (GPAQ) in assessing levels and change in moderate-vigorous physical activity and sedentary behaviour. BMC Public Health. 2014;14:1255. 25492375 10.1186/1471-2458-14-1255 PMC4295403

[24] Du Y, Liu B, Sun Y, et al. Trends in adherence to the physical activity guidelines for Americans for aerobic activity and time spent on sedentary behavior among US adults, 2007 to 2016. JAMA Netw Open. 2019;2(7):e197597. 31348504 10.1001/jamanetworkopen.2019.7597 PMC6661709

[25] Statistics NCfH. Continuous NHANES public-use linked mortality files, 2019. 2022.

[26] Harrell FE. General aspects of fitting regression models. Regression modeling strategies: with applications to linear models, logistic and ordinal regression, and survival analysis. Cham: Springer; 2015. p. 13–44.

[27] Schneider B. Analysis of clinical trial outcomes: alternative approaches to subgroup analysis. Control Clin Trials. 1989;10(4 Suppl):176S–186S. 2605966 10.1016/0197-2456(89)90056-1

[28] Imai K, Keele L, Tingley D. A general approach to causal mediation analysis. Psychol Methods. 2010;15(4):309–334. 20954780 10.1037/a0020761

[29] Bach KE, Kelly JT, Palmer SC, et al. Healthy dietary patterns and incidence of CKD: a meta-analysis of cohort studies. Clin J Am Soc Nephrol. 2019;14(10):1441–1449. 31551237 10.2215/CJN.00530119 PMC6777603

[30] Sahni N, Gupta KL, Rana SV, et al. Intake of antioxidants and their status in chronic kidney disease patients. J Ren Nutr. 2012;22(4):389–399. 22227184 10.1053/j.jrn.2011.09.002

[31] Alehagen U, Aaseth J, Alexander J, et al. Selenium and coenzyme Q10 supplementation improves renal function in elderly deficient in selenium: observational results and results from a subgroup analysis of a prospective randomised double-blind placebo-controlled trial. Nutrients. 2020;12(12):3780. 33317156 10.3390/nu12123780 PMC7764721

[32] Xie C, Zeng M, Shi Z, et al. Association between selenium status and chronic kidney disease in middle-aged and older Chinese based on CHNS data. Nutrients. 2022;14(13):2695. 35807874 10.3390/nu14132695 PMC9269073

[33] Sies H, Jones DP. Reactive oxygen species (ROS) as pleiotropic physiological signalling agents. Nat Rev Mol Cell Biol. 2020;21(7):363–383. 32231263 10.1038/s41580-020-0230-3

[34] Himmelfarb J, Ikizler TA, Ellis C, et al. Provision of antioxidant therapy in hemodialysis (PATH): a randomized clinical trial. J Am Soc Nephrol. 2014;25(3):623–633. 24371300 10.1681/ASN.2013050545 PMC3935590

[35] Jun M, Venkataraman V, Razavian M, et al. Antioxidants for chronic kidney disease. Cochrane Database Syst Rev. 2012;10(10):Cd008176. 23076940 10.1002/14651858.CD008176.pub2 PMC8941641

[36] Rapa SF, Iorio D, Campiglia BR, et al. S. Inflammation and oxidative stress in chronic kidney disease-potential therapeutic role of minerals, vitamins and plant-derived metabolites. Int J Mol Sci. 2019;21(1):263. 10.3390/ijms21010263 PMC6981831 31906008

[37] Sporn MB, Liby KT, Yore MM, et al. New synthetic triterpenoids: potent agents for prevention and treatment of tissue injury caused by inflammatory and oxidative stress. J Nat Prod. 2011;74(3):537–545. 21309592 10.1021/np100826q PMC3064114

[38] Pergola PE, Raskin P, Toto RD, et al. Bardoxolone methyl and kidney function in CKD with type 2 diabetes. N Engl J Med. 2011;365(4):327–336. 21699484 10.1056/NEJMoa1105351

[39] Nangaku M, Kanda H, Takama H, et al. Randomized clinical trial on the effect of bardoxolone methyl on GFR in diabetic kidney disease patients (TSUBAKI study). Kidney Int Rep. 2020;5(6):879–890. 32518870 10.1016/j.ekir.2020.03.030 PMC7271944

[40] Kanlaya R, Thongboonkerd V. Protective effects of epigallocatechin-3-Gallate from green tea in various kidney diseases. Adv Nutr. 2019;10(1):112–121. 30615092 10.1093/advances/nmy077 PMC6370267

[41] Maleki SJ, Crespo JF, Cabanillas B. Anti-inflammatory effects of flavonoids. Food Chem. 2019;299:125124. 31288163 10.1016/j.foodchem.2019.125124

[42] Abbasi M, Daneshpour MS, Hedayati M, et al. Dietary total antioxidant capacity and the risk of chronic kidney disease in patients with type 2 diabetes: a nested case-control study in the Tehran Lipid Glucose Study. J Ren Nutr. 2019;29(5):394–398. 30709711 10.1053/j.jrn.2018.11.008

[43] Moludi J, Tandorost A, Kamari N, et al. Dietary total antioxidant capacity and its association with renal function and kidney stones: results of a RaNCD cohort study. Food Sci Nutr. 2022;10(5):1442–1450. 35592299 10.1002/fsn3.2753 PMC9094466

[44] Gansevoort RT, Correa-Rotter R, Hemmelgarn BR, et al. Chronic kidney disease and cardiovascular risk: epidemiology, mechanisms, and prevention. Lancet. 2013;382(9889):339–352. 23727170 10.1016/S0140-6736(13)60595-4

[45] Jovanovich A, Isakova T, Stubbs J. Microbiome and cardiovascular disease in CKD. Clin J Am Soc Nephrol. 2018;13(10):1598–1604. 29743160 10.2215/CJN.12691117 PMC6218820

[46] de Zeeuw D, Akizawa T, Audhya P, et al. Bardoxolone methyl in type 2 diabetes and stage 4 chronic kidney disease. N Engl J Med. 2013;369(26):2492–2503. 24206459 10.1056/NEJMoa1306033 PMC4496027

[47] Rossing P, Block GA, Chin MP, et al. Effect of bardoxolone methyl on the urine albumin-to-creatinine ratio in patients with type 2 diabetes and stage 4 chronic kidney disease. Kidney Int. 2019;96(4):1030–1036. 31377056 10.1016/j.kint.2019.04.027

[48] Rysz J, Franczyk B, Ławiński J, et al. The impact of CKD on uremic toxins and gut microbiota. Toxins. 2021;13(4):252. 33807343 10.3390/toxins13040252 PMC8067083

[49] Zeng Y, Guo M, Fang X, et al. Gut microbiota-derived trimethylamine N-oxide and kidney function: a systematic review and meta-analysis. Adv Nutr. 2021;12(4):1286–1304. 33751019 10.1093/advances/nmab010 PMC8321840

[50] Lano G, Burtey S, Sallée M. Indoxyl sulfate, a uremic endotheliotoxin. Toxins. 2020;12(4):229. 32260489 10.3390/toxins12040229 PMC7232210

[51] Ravid JD, Kamel MH, Chitalia VC. Uraemic solutes as therapeutic targets in CKD-associated cardiovascular disease. Nat Rev Nephrol. 2021;17(6):402–416. 33758363 10.1038/s41581-021-00408-4

[52] Ranganathan N, Anteyi E. The role of dietary fiber and gut microbiome modulation in progression of chronic kidney disease. Toxins. 2022;14(3):183. 35324680 10.3390/toxins14030183 PMC8955792

[53] Yang X, Soohoo M, Streja E, et al. Serum magnesium levels and hospitalization and mortality in incident peritoneal dialysis patients: a cohort study. Am J Kidney Dis. 2016;68(4):619–627. 27261330 10.1053/j.ajkd.2016.03.428 PMC5035573

[54] Leenders NHJ, Vervloet MG. Magnesium: a magic bullet for cardiovascular disease in chronic kidney disease? Nutrients. 2019;11(2):455. 30813254 10.3390/nu11020455 PMC6412491

[55] Ferrè S, Li X, Adams-Huet B, et al. Association of serum magnesium with all-cause mortality in patients with and without chronic kidney disease in the Dallas Heart Study. Nephrol Dial Transplant. 2018;33(8):1389–1396. 29077944 10.1093/ndt/gfx275 PMC6454476

