# Supplementary material for: Association of dietary total antioxidant capacity with all-cause and cardiovascular mortality in patients with chronic kidney disease: based on two retrospective cohort studies of NHANES
Source: Ren Fail. 2023 May 31;45(1):2205950. doi: 10.1080/0886022X.2023.2205950 (PMC10236956; doi:10.1080/0886022X.2023.2205950)
Supplement: Supplemental Material [file IRNF_A_2205950_SM5129.pdf]

Supplement TABLE1.Descriptive characteristics by the Vitamin C Equivalent Antioxidant Capacity (VCEAC) among adults with CKD in NHANES (2007-2018).

Variable	VCEAC Q1 (N=1582)	Q2 (N=1583)	Q3 (N=1583)	Q4 (N=1582)	*P* Value[b]
**VCEAC**	＜137.21	137.21,280.93	280.93,534.87	≥534.87	
**Age,year,Mean(SD)**	58.58(15.72)	59.86(15.25)	61.41(14.05)	61.44(14.20)	0.012
**Sex,n(%)**					
Female	745(24.7)	783(25.9)	739(24.5)	753(24.9)	0.412
Male	837(25.3)	800(24.2)	844(25.5)	829(25.0)	
**Ethnicity, n (%)**					
Mexican American	201(30.0)	197(29.4)	150(22.4)	121(18.1)	
White	731(20.5)	886(24.8)	970(27.2)	981(27.5)	
Black	354(31.1)	277(24.3)	256(22.5)	252(22.1)	<0.001
Latin	172(33.0)	127(24.4)	119(22.8)	103(19.8)	
Other	124(28.6)	96(22.2)	88(20.3)	125(28.9)	
**Education,n(%)**					
Grades 0-12	1574(24.9)	1579(25.0)	1582(25.1)	1580(25.0)	
High school graduate/GED	3(33.3)	3(33.3)	1(11.1)	2(22.2)	0.05
College or above	5(83.3)	1(16.7)	0(0.0)	0(0.0)	
**Income, n (%)**					
Under $75,000	1265(27.1)	1197(25.6)	1132(24.2)	1076(23.0)	
$75,000 and over	236(17.2)	299(21.8)	389(28.4)	448(32.7)	<0.001
Other	81(28.1)	87(30.2)	62(21.5)	58(20.1)	
**smoking, n (%)**					
Yes	857(27.3)	813(25.9)	758(24.1)	714(22.7)	<0.001
No	718(22.6)	766(24.1)	823(25.9)	865(27.3)	
**Hypertension,n(%)**					
Yes	830(24.9)	849(25.4)	838(25.1)	823(24.6)	0.590
No	749(25.1)	730(24.5)	742(24.9)	759(25.5)	
**Diabetes**					
Yes	369(26.5)	350(25.1)	359(25.8)	315(22.6)	0.234
No	1212(24.6)	1233(25.0)	1224(24.8)	1266(25.7)	
**CVD**					
Yes	349(26.5)	348(26.4)	323(24.5)	297(22.6)	0.140
No	1212(24.5)	1221(24.7)	1247(25.2)	1270(25.7)	
**Cancer**					
Yes	195(18.5)	252(23.9)	302(28.6)	307(29.1)	<0.001
No	1375(26.2)	1326(25.3)	1274(24.3)	1272(24.2)	
**Alcohol,g,Mean(SD)**	15.43(37.03)	14.35(34.89)	14.39(34.76)	16.38(35.42)	<0.001
**Protein,g,Mean(SD)**	121.08(50.51)	149.05(60.60)	159.65(60.94)	178.00(73.02)	<0.001

Information Classification: General

**Energy intake(kcal/day), Mean (SD)**	3164.10(1226.19)	3803.00(1472.53)	4050.18(1467.05)	4407.37(1718.82)	<0.001
**Carbohydrate,g,Mean(SD)**	373.62(162.83)	453.23(189.50)	487.71(192.34)	536.94(222.69)	<0.001
**Dietary fiber,g,Mean(SD)**	20.35(10.27)	28.90(13.17)	35.25(15.53)	44.36(18.79)	<0.001
**Total fat,g,Mean(SD)**	123.02(57.97)	148.93(70.66)	157.91(69.84)	168.02(81.71)	<0.001
**Vitamin A,ug,Mean(SD)**	589.77(353.28)	956.44(520.33)	1241.68(686.06)	2025.93(1035.70)	<0.001
**Vitamin C,mg,Mean(SD)**	77.57(73.78)	137.48(111.99)	170.47(131.61)	242.91(175.74)	<0.001
**Vitamin E,mg,Mean(SD**	10.10(6.04)	13.52(7.89)	16.26(8.75)	20.14(10.99)	<0.001
**Beta-carotene,ug,Mean(SD)**	537.18(390.44)	1490.21(871.66)	3630.00(2018.56)	11177.09(8233.14)	<0.001
**Alpha-carotene, ug,Mean(SD)**	57.43(77.25)	207.80(241.65)	581.27(584.70)	2222.72(2818.08)	<0.001
**Beta-cryptoxanthin ,ug,Mean(SD)**	64.00(96.57)	133.46(174.32)	188.03(291.49)	291.24(789.74)	<0.001
**Lycopene,ug,Mean(SD)**	1927.93(2325.83)	5559.22(5362.03)	10360.61(10405.50)	15962.97(20362.41)	<0.001
**Lutein+zeaxanthin, ug,Mean(SD)**	756.17(487.76)	1599.83(1122.70)	2763.16(2170.13)	6300.02(7470.68)	<0.001
**Magnesium,mg,Mean(SD)**	—	—	—	—	—
**Selenium,ug,Mean(SD)**	—	—	—	—	—
**Zinc,mg,Mean(SD)**	—	—	—	—	—
**29 dietary flavonoids , mg, Mean (SD)**	42.55(72.49)	125.10(192.24)	242.68(355.80)	388.21(688.23)	<0.001
**BMI,kg/m²,Mean(SD)**	29.49(6.69)	29.28(6.08)	29.16(6.03)	28.56(5.83)	0.001
**CKD_EPI_Scr, ml/min / 1.73 m^2,Mean（SD）**	73.10(16.71)	73.03(15.99)	73.35(14.88)	73.99(13.50)	<0.001
**Urine Albumin creatinine ratio ,mg/g,Mean(SD)**	76.97(322.57)	68.67(365.54)	54.80(234.47)	40.24(173.12)	<0.001
**PA_total_MET,mins/wk,Mean(SD)**	4683.32(7562.16)	4170.77(5752.51)	3942.60(5823.22)	4045.49(5789.74)	0.734

***Note*：**

Percentages may not total 100 because of rounding. NA=not applicable

VCEAC = Vitamin C Equivalent Antioxidant Capacity. Q = quartile; CVD, cardiovascular disease; BMI = body mass index; MET, metabolic equivalents. Summary statistics for independent variables were calculated as means and standard deviation (SD) or case numbers and percentages for categorical variables. The unit for VCEAC was vitamin C equivalents per 100 g.

[b] *P* value was calculated by linear model for continuous variables and chi-square test for categorical variables.

Information Classification: General

Supplement TABLE2.Descriptive characteristics by the Component Dietary Antioxidant Index (CDAI) among adults with CKD in NHANES (2007-2018).

Variable	CDAI Q1 (N=1575)	Q2 (N=1575)	Q3 (N=1575)	Q4 (N=1575)	P Value[b]
**CDAI**	Q1＜-7.72	-7.72≤Q2＜-1.78	-1.78≤Q3＜8.33	Q4≥8.33	
**Age,year,Mean(SD)**	63.36(14.46)	63.03(14.88)	62.21(14.79)	62.33(14.01)	0.012
**Sex,n(%)**					
Female	868（28.8）	767（25.5）	709（23.6）	665（22.1）	<0.001
Male	707（21.5)	808(24.6)	866(26.3)	910(27.7)	
**Ethnicity, n (%)**					
Mexican American	187(28.0)	199(29.8)	161(24.1)	121(18.1)	<0.001
White	776(21.9)	861(24.3)	921(26.0)	991(27.9)	
Black	169(32.4)	138(26.5)	126(24.2)	88(16.9)	
Latin	366(32.2)	285(25.1)	251(22.1)	233(20.5)	
Other	77(18.0)	92(21.5)	116(27.2)	142(33.3)	
**Education,n(%)**					
Grades 0-12	1570（25）	1571（25）	1570（25）	1574（25）	0.600
High school graduate/GED	2（22.2）	3（33.3）	3（33.3）	1（11.1）	
College or above	3（50）	1（16.7）	2（33.3）	0（0）	
**Income, n (%)**					
Under $75,000	1288（27.7）	1197（25.8）	1143（24.6）	1020（21.9）	<0.001
$75,000 and over	198（14.5）	307（22.5）	370（27.1）	489（35.9）	
Other	89（30.9）	71（24.7）	62（21.5）	66（22.9）	
**smoking, n (%)**					
Yes	849(27.2)	800(25.6)	760(24.3)	713(22.8)	<0.001
No	721(22.8)	772(24.4)	810(25.6)	859(27.2)	
**Hypertension,n(%)**					
Yes	909(27.3)	861(25.9)	797(24.0)	757(22.8)	<0.001
No	662(22.3)	713(24.0)	774(26.1)	817(27.5)	
**Diabetes**					
Yes	377(27.2)	397(28.7)	335(24.2)	276(19.9)	<0.001
No	1198(24.4)	1177(24.0)	1240(25.2)	1298(26.4)	
**CVD**					
Yes	407(31.0)	328(25.0)	301(22.9)	278(21.2)	<0.001
No	1154(23.4)	1228(24.9)	1259(25.6)	1282(26.0)	
**cancer**					
Yes	223(21.2)	259(24.6)	269(25.5)	303(28.7)	0.002
No	1345(25.8)	1309(25.1)	1298(24.9)	1267(24.3)	
**Alcohol,g,Mean(SD)**	09.00(27.27)	14.45(33.57)	16.42(36.42)	19.59(40.51)	<0.001
**Protein,g,Mean(SD)**	113.74(39.85)	149.17(50.33)	167.72(62.90)	184.67(75.51)	<0.001

Information Classification: General

**Energy intake(kcal/day), Mean (SD)**	2937.87(1029.83)	3799.25(1282.97)	4219.13(1467.84)	4622.24(1751.27)	<0.001
**Carbohydrate,g,Mean(SD)**	353.19(145.21)	454.46(175.25)	506.40(182.35)	558.11(230.15)	<0.001
**Dietary fiber,g,Mean(SD)**	20.39(09.01)	29.85(11.37)	37.12(13.98)	45.15(20.40)	<0.001
**Total fat,g,Mean(SD)**	115.22(50.40)	147.87(62.59)	163.29(75.00)	177.65(82.20)	<0.001
**Vitamin A,ug,Mean(SD)**	/	/	/	/	
**Vitamin C,mg,Mean(SD)**	76.62(64.99)	130.50(87.48)	192.41(130.35)	248.20(176.47)	<0.001
**Vitamin E,mg,Mean(SD**	09.53(04.63)	13.53(06.04)	16.88(08.40)	21.29(11.86)	<0.001
**Beta-carotene ,ug,Mean(SD)**	1539.37(1802.14)	3358.23(3388.50)	5183.37(4962.27)	8295.61(8916.95)	<0.001
**Alpha-carotene ,ug,Mean(SD)**	272.50(485.85)	580.52(887.75)	1003.29(1350.60)	1535.15(2865.28)	<0.001
**Beta-cryptoxanthin ,ug,Mean(SD)**	70.31(100.12)	132.09(166.85)	198.75(263.75)	299.78(786.18)	<0.001
**Lycopene,ug,Mean(SD)**	4657.64(6624.23)	8269.50(10109.23)	10551.17(13116.74)	13270.60(18687.35)	<0.001
**Lutein+zeaxanthin, ug, Mean(SD)**	1236.42(1390.99)	2226.22(2483.70)	3285.82(3567.38)	5466.15(7251.35)	<0.001
**Magnesium,mg,Mean(SD)**	386.10(121.12)	530.83(144.37)	624.86(186.11)	754.90(288.20)	<0.001
**Selenium,ug,Mean(SD)**	156.74(60.14)	203.97(73.42)	230.88(97.16)	251.67(111.99)	<0.001
**Zinc,mg,Mean(SD)**	15.44(06.30)	20.89(08.40)	24.52(11.30)	28.21(18.77)	<0.001
**29 dietary flavonoids ,mg,Mean(SD)**	20.70(31.14)	53.59(64.51)	130.75(140.12)	540.60(509.39)	<0.001
**BMI,kg/m^2,Mean(SD)**	29.58(06.41)	29.31(06.12)	28.81(05.89)	28.63(05.91)	<0.001
**CKD_EPI_Scr, ml/min / 1.73 m^2,Mean（SD）**	71.54(16.79)	73.23(15.53)	74.45(14.62)	74.08(13.42)	<0.001
**Urine Albumin creatinine ratio ,mg/g,Mean(SD)**	78.58(345.05)	65.72(299.52)	45.07(169.14)	45.75(273.18)	<0.001
**PA_total_MET, mins/wk,Mean(SD)**	4053.16(6649.48)	4092.24(6091.53)	4312.40(6100.44)	4076.34(5613.41)	0.003

***Note*：**

Percentages may not total 100 because of rounding. NA=not applicable

CDAI = Component Dietary Antioxidant Index; CVD, cardiovascular disease; Q = quartile; BMI = body mass index; MET, metabolic equivalents. Summary statistics for independent variables were calculated as means and standard deviation (SD) or case numbers and percentages for categorical variables. The CDAI with negative values was computed from standardized antioxidant intake and principal component analysis and had no unit. The unit for VCEAC was vitamin C equivalents per 100 g.

[b] *P* value was calculated by linear model for continuous variables and chi-square test for categorical variables.

Information Classification: General

Supplement TABLE3. Sensitivity Analyses of Multi-variable Adjusted Association Between DTAC and mortality

	All-cause mortality		CVD mortality	
	HR (95% CI)	*P* Value	HR (95% CI)	*P* Value
**VCEAC**				
Model1	0.99(0.98-1.01)	0.306	0.99(0.98-1.01)	0.355
Model2[a]	0.99(0.97-1.02)	0.525	0.99(0.96-1.03)	0.765
Model3 [b]	0.99(0.97-1.01)	0.352	0.98(0.95-1.02)	0.341
Model4[c]	0.99(0.96-1.02)	0.535	0.98(0.95-1.02)	0.347
Model5[d]	1.02(0.99-1.04)	0.152	1.01(0.97-1.05)	0.727
**CDAI**				
Model1	0.98(0.95-1.01)	0.230	1.01(0.96-1.05)	0.775
Model2[a]	0.98(0.94-1.01)	0.146	1.00(0.95-1.06)	0.909
Model3[b]	0.98(0.95-1.01)	0.194	1.01(0.96-1.05)	0.784
Model4[c]	1.00(0.96-1.03)	0.785	1.00(0.95-1.05)	0.967
Model5[d]	1.01(0.97-1.05)	0.706	1.00(0.94-1.07)	0.946

Notes:

HR=Hazard ratio; CI, confidence intervals; CVD, cardiovascular disease; VCEAC, Vitamin C Equivalent Antioxidant Capacity. CDAI, Component Dietary Antioxidant Index.

Model1: Adjusted for age, Sex, ethnicity, income, and education level, potassium intake, protein intake, carbohydrates intake, dietary fiber intake, total fat intake, alcohol intake, total energy intake was adjusted via the residual method, and smoking, and MET-PA, diabetes, hypertension, CVD and cancer, urine albumin, eGFR, BMI.

Model2[a]: Model1 excluded participants whose cause of death is trauma.

Model3[b]: Model1 excluded participants who died having less than two years of follow-up.

Model4[c]: Model1 excluded participants who died of malignant neoplasms.

Model5[d]: Model1 excluded participants with mild CKD (1-2 stages).

Information Classification: General

Supplementary Methods

Calculation of the CDAI and evaluation of dietary total antioxidant capacity

The data came from the National Health and Nutrition Examination Survey (NHANES) Dietary information, including two 24-hour recalls and an food frequency questionnaire (FFQ). Participants reported foods/drinks consumed in 24 hours. The questionnaire can be found at https://epi.grants.cancer.gov/diet/usualintakes/ffq.html.

To calculate dietary total antioxidant capacity (VCEAC), the daily intake of each antioxidant was multiplied by its antioxidant capacity (VCE) and then summed. Antioxidant vitamins (vitamin C, vitamin E, carotenoids) and other antioxidants (flavonoids, isoflavones, proanthocyanidins) were calculated from dietary recall data linked to an expanded flavonoid database[1-3]. The antioxidant capacity of vitamin C, 29 types of flavonoids, 5 types of carotenoids, vitamin A, and vitamin E was analyzed using the 2,2'-azino-bis(3-ethylbenzthiazoline) 6-sulphonic acid (ABTS) method[4]. Minerals (magnesium, selenium, and zinc) were excluded from VCEAC calculation as their antioxidant capacity cannot be tested by the ABTS method. The VCEAC assay used a vitamin C standard curve to relate vitamin C concentration to absorbance reduction at 734nm. The higher the VCEAC value of a compound, the more effective its antioxidant activity.

The calculation of VCEAC of each compound was made using vitamin C standard curve (Equation 1) as follows:

$$\text{VCEAC (mg/L)} = (\Delta\ \text{Abs} - a)/b \quad (1)$$

where a: y-intercept of vitamin C standard curve

b: slope of vitamin C standard curve

Δ Abs: the initial absorbance of control minus the resulting absorbance of chemicals tested at 10 min, 734 nm and 37◦C.

For CDAI calculation, the intake of selected antioxidants (including 29 types of flavonoids, 5 types of carotenoids, vitamin A, vitamin C, vitamin E, and 3 types of minerals as above) was normalized by the zero-mean method [(daily intake of antioxidant - mean intake of antioxidant)/standard deviation]. Then the total

Information Classification: General

information of all antioxidant categories was summed as previously described[5,6].

We used the residual method to control for confounding bias. The residual method involves fitting a multiple regression model with the outcome as the dependent variable and the exposure variable(s) and potential confounding variables as independent variables. The residuals from this model are then used as a new variable in subsequent analyses to control for confounding factors. By including these residuals in subsequent analyses, we can adjust for the effects of confounding factors and obtain more accurate estimates of the association between exposure and outcome.

References

1. Bhagwat S, Haytowitz D, Prior R, et al. USDA database for proanthocyanidin content of selected foods. 2004.

2. Bhagwat S, Haytowitz DB, Holden JMJMUDoA. USDA database for the isoflavone content of selected foods, release 2.0. 2008; **15**.

3. Bhagwat S, Haytowitz DB, Holden JMJUDoAB, MD, USA. USDA database for the flavonoid content of selected foods, Release 3.1. 2014.

4. Floegel A, Kim D-O, Chung S-J, et al. Development and validation of an algorithm to establish a total antioxidant capacity database of the US diet. 2010; **61**(6): 600-23.

5. Wright ME, Mayne ST, Stolzenberg-Solomon RZ, et al. Development of a comprehensive dietary antioxidant index and application to lung cancer risk in a cohort of male smokers. 2004; **160**(1): 68-76.

6. Yu YC, Paragomi P, Wang R, et al. Composite dietary antioxidant index and the risk of colorectal cancer: Findings from the Singapore Chinese Health Study. 2022; **150**(10): 1599-608.

Information Classification: General
